# The 43rd Vernon‐Wall Lecture: What working relationally brings to problem‐solving

**DOI:** 10.1111/bjep.12771

**Published:** 2025-03-31

**Authors:** Anne Edwards

**Affiliations:** ^1^ Department of Education University of Oxford Oxford UK

**Keywords:** collaboration, common knowledge, cultural‐historical, interprofessional, relational agency, relational expertise

## Abstract

**Aim:**

Responses to complex problems demand collaboration across practice boundaries. The studies presented here make visible the processes that constitute successful collaborations between practitioners and clients.

**The Argument:**

Complex problems can be *discovered*, where practitioners recognize and address complexity through an existing repertoire of responses. Or they are *created* problems, where researchers reveal new phenomena and create new knowledge. That knowledge can then be used to inform responses to discovered problems.

**The Studies:**

The research on created problems discussed here asked: What happens at the intersection of practices during work on complex problems; how are motives aligned to expand and respond to the problem; and what kind of expertise is involved? The discovered problems were in: pedagogic work with parents in a daycare centre, the development of an app to support autistic people in the workplace and an evaluation framework examining interprofessional working.

**The Relational Concepts:**

New knowledge, arising in studies of emergent interprofessional working in English children's services, explains how working relationally can strengthen responses. The three relational concepts are based in cultural‐historical approaches to human development. They are: *relational expertise,* which builds *common knowledge*, comprising the motives of participants, which mediates collaborations, creating *relational agency.* Relational agency then strengthens the actions taken by practitioners and clients.

**Implications:**

The relational concepts offer guidance for training practitioners in interprofessional working and in building the agency of clients. They offer researchers a framework for examining collaborations and show how cultural‐historical research methods capture concepts in practitioners' actions.

## INTRODUCTION

Most professional interactions involve relational work, both with other practitioners and with clients. These relational interactions are not the female emotional labour described in Hochschild's (1983) seminal work. Rather, they mark a growing awareness among professionals such as trauma specialists that working relationally is a crucial component in their professional repertoires (Saakvitne, 2017). For educators, psychologists and others in the caring professions, successful relationship building frequently involves invisible backstage work (De Frino, 2009; Fletcher, 2001), where trust is built and employed. The argument to be taken forward in this article is that this invisible work can be made visible, the rationales for strong collaborations understood and ways of accomplishing cooperation made clear.

Professional collaboration comes in many forms, within and across established teams. It includes the interdependency of responsive nursery‐school workers; the quick reactions of emergency services; and the alignment of interprofessional efforts to support a troubled family. When successful, these interactions may be marked by a sense of mutuality, an alertness to what the other person is likely to do and an awareness of why they might do it. But we also know that collaborations can fall short of these features and fail to take advantage of the specialist expertise available. A classic example is an analysis of a case conference about a troubled child, where respect for the power of the psychologist and a hidden aim of securing additional funding for the school, meant that the knowledge and expertise of the child's class teacher and parent were ignored to the detriment of the child (Hjorne & Säljö, 2004).

The examples of relational collaboration to be discussed here have ensured that a wide range of specialist expertise is brought to bear to both interpret and respond to a problem, such as the life trajectory of a troubled child. The examples do not involve heroic boundary crossing, where a practitioner enters the alien practices of another profession. Rather, the focus is the small steps that can be taken to ensure that the most relevant expertise is employed in interpreting and responding to complex problems. That is, what working relationally looks like.

The problems to be addressed by practitioners can be classified in Getzels' terms as *Discovered* and *Created* problems (Getzels, 1979). The former are typical of demanding situations met by practitioners in everyday life, where there are known ways of tackling them. The latter are typical of newly arisen complex problems to be worked on by professionals and researchers. In created problems, this complexity is sought, interpreted and described. Simply describing such problems can lead to new knowledge, while the processes involved in recognizing and interpreting them can inform new ways of tackling them. Discovered problems are likely to be found in professional work with clients where practitioners have a pedagogic role and in teamwork, where roles and their inter‐relationships are well understood. Created problems are, for example, to be found in new collaborations between practitioners and in multidisciplinary research, where working together leads to new understandings of a problem and may call for new ways of operating professionally.

I shall use examples of interprofessional work in children's services to reveal the processes involved in working relationally on created problems and then discuss examples of how these processes were used in discovered problems in research, practice and evaluation. The focus will therefore be on the small steps that comprise relational work and the benefits that can accrue.

## METHODOLOGICAL IMPLICATIONS OF CREATED PROBLEMS

Created problems, if they are to expand understandings of new phenomena, need to employ the widest range of possible interpretations and responses. This requires researchers to also take a relational approach (Hasse, 2017). We need to be alongside those taking forward new ways of interpreting and responding in order to conversationally access their insights and concerns, to share our ongoing analyses and to respond to their interpretations of these analyses. In brief, these problems demand a reflexive participatory approach to research, where researchers position themselves as part of the field.

Examples of such engaged close‐to‐practice research within the broad cultural‐historical field include Hedegaard's groundbreaking work on studying young children (Hedegaard & Fleer, 2008). There her analyses of young children in activities includes details of the part she has played in the settings. Jornet (2023) more recently has argued that we should follow Dewey (1929) by avoiding a ‘spectator view of knowledge’ (p. 23). To prove his point, he offers a rich explanation of how becoming a participant observer in a primary school's arts‐based curriculum, rather than a spectator of the activities, reminds us that research can include activism aimed at overcoming disadvantage. There are many other examples of such engaged research within the broad Cultural‐Historical Activity Theory (CHAT) field (Yamagata‐Lynch, 2010). These include Engeström's Developmental Work Research (DWR) (Engeström, 2005), Stetsenko's Transformative Activist Stance (Stetsenko, 2017) and Sannino's Transformative Agency through Double Stimulation (Sannino, 2020).

These approaches align with my concern with research on created problems. Wardekker (2000) showed clearly why the different criteria for quality employed by nomological or even most interpretative approaches to research cannot capture the processes of becoming within practices, which are themselves located within wider cultural dynamics. Instead, he argued that ‘…the product of research is not knowledge in the sense of a product that can be transferred to other persons and situations; it is an understanding of the change processes in a specific situation that may or may not have implications for other situations.’ (p. 269).

The created problem, what is involved in successful interprofessional work, was explored between 2002 and 2006 in two parallel studies: Learning in and For Interagency Working (LIW) (Edwards et al., 2009) and the National Evaluation of the Children's Fund (NECF) (Edwards et al., 2006). Both studies, with ethical approval from the University of Birmingham, examined new ways of collaborating across the practice boundaries of the professions that were contributing to children's services in Local Authorities in England. Promoting interprofessional, or interagency working, arose from the government aim to prevent debilitating social exclusion and to focus on the wellbeing of children and young people. The timing of the policy roll‐out and the varied settings across Local Authorities required us to study these new phenomena while they were in development, which meant we could characterize our research focus as a created problem.

The research methods used in the analyses to be discussed here were observations of interprofessional meetings, interviews and DWR interventionist workshops. These structured and recorded workshops required the participating practitioners to use the conceptual tools of activity theory to interrogate their everyday practices, for example, expanding their understanding of the problem they are working on (Engeström, 2005). Further details of methods and analyses are to be found in the full account of the LIW study (Edwards et al., 2009).

## THE THEORETICAL BACKGROUND

Problem‐solving professional work rarely involves unbending ritualized patterns of learnt behaviour. Even with discovered problems, it comprises thoughtful decision‐making which is responsive to the situation. The conceptual tools for taking small steps in relational collaborations are offered in this article as resources for explaining and encouraging such thoughtful engagement, but not as blueprints. Engeström's declaration: ‘My key message is that forming theoretical concepts is more important than ever in our age of chaotic complexity and crises. People need instruments that allow them to grasp and redirect societal processes that are increasingly out of control ….’ (Engeström, 2020, pp. 32) accords with the line I am taking. My aim in developing theoretical concepts is to clarify the processes and associated expertise that makes for productive collaborations, so that they can inform professional repertoires of action. Wardekker's view that: ‘Knowledge is a mediational means for focusing our attention on specific aspects of practice.’ (Wardekker, 2000, p. 269) captures these intentions.

First some conceptual ground clearing. The analyses to be discussed are based in cultural‐historical theory, initiated by Vygotsky in the 1920s and early 30s in Russia (Vygotsky, 1997a, 1997b). Vygotsky's work has, in particular, alerted us to the dialectical relationship between a person and the worlds they inhabit, that underpins human learning. These ideas were first taken forward by his students and their colleagues in Russia (Galperin, 1969; Davydov, 1988–1989); (see also Engeness and Lund (2020) for a recent reading of Galperin's work). The ideas are now being developed in international research across the CHAT field.

A cultural‐historical perspective sees concepts as tools for taking action in the world. Concepts emerge as they are used in practices and both shape and are shaped by the demands and opportunities embedded in practices. This understanding of practices recognizes that they are created by the actions of those who participate in them; while the knowledge and values that signify a practice become segmented over time. In brief, practices are ‘…historically accumulated, knowledge‐laden, emotionally freighted and given direction by what is valued by those who inhabit them.’ (Edwards, 2010, p.7). Despite the segmentation that can lead to strong boundaries between different professional practices, practices do mutate as new ideas and technological changes mediate what is focused on and how it is tackled.

We therefore now turn to the focus of activities in practices and how that focus is approached in the development of Vygotsky's groundwork by Leont'ev. Here I draw only on his notion of the object of activity and the associated object motive (Leont'ev, 1978). According to Leont'ev, the idea of the object of activity allows us to objectify what is worked on in an activity, the problem. How the problem is interpreted and acted on is explained by his idea of the object motive. The object motive comprised the needs, emotions and feelings a person associates with the object of activity. For example, when discussing a troubled child, a psychologist may focus on mental health and what they could do, while a social worker may first consider family dynamics and how they might take professional action.

Hedegaard has more recently built on Leont'ev's work to recognize the importance of practices in the development of object motives. Her idea of motive orientation (Hedegaard, 2012) explains how people are oriented to aspects of activities or problems in ways that reflect their prior experiences, their values and where they think they can be effective. There is therefore a strong affective element in practitioners' orientations, alongside their professional knowledge. In summary, how a practitioner interprets and responds to a problem, such as the trajectory of a troubled child, is also mediated by their motive orientation, what they bring from their experience in the practices of, for example, educational psychology or social work.

Importantly, professionals from different practices will bring different motive orientations to a problem. While collaborating in those circumstances can be challenging, it also offers the opportunity to expand interpretations of an object of activity, such as a child's trajectory, and offers a wide range of informed responses to the problem.

While studying interprofessional work with troubled children in England in the LIW and NECF projects, I attempted to answer the following questions:

- What happens at the intersection of practices when people work on complex problems?
- How are motive orientations aligned so that the object of activity is expanded and interprofessional work can be accomplished?
- What kind of expertise is involved in this process?

## WORKING RELATIONALLY

The three conceptual tools employed in successful interprofessional working were first recognized in an earlier study of pedagogic work between family workers and their clients, aimed at strengthening the agency of the client (Edwards, 2005). The resources were then refined in LIW and NECF. The concepts are: relational expertise, common knowledge and relational agency (Edwards, 2005, 2010, 2012, 2017). Relational expertise is a form of expertise that is in addition to the specific expertise of, for example, a psychologist. It involves (i) recognizing the standpoints and the motive orientations to the problem held by those who inhabit other practices; and (ii) being clear about one's own standpoints and motive orientations. The exercise of relational expertise leads to the creation of common knowledge.

Common knowledge comprises the motive orientations of all participants. It is not the knowledge of another person's job, rather it consists of an understanding of how and why different participants are orienting to a problem. It is built using relational expertise to first recognize similar long‐term broad goals, such as children's wellbeing and then to reveal categories, values and motives in talk about the problem. Here is an educational psychologist in a DWR workshop in the LIW study explaining the need for common knowledge. ‘I think the very first step is understanding about what the sort of issues are….Professions have very, very different ideas about need, about discipline, about responsibility, about the impact of systems on families…So I think the first step is actually to get some shared understanding about effective practices and about understanding the reasons behind some of them’.

This common knowledge then mediates joint action on a problem, first while interpreting it and then while responding to the expanded interpretation. This joint action, the unfolding of relational agency, leads to deeper understandings of a complex problem and strengthens professional responses to it. In summary, in interprofessional work relational agency involves: (i) expanding the object of activity by recognizing the object motives of others as they interpret the problem; and (ii) aligning one's own responses to the enhanced interpretations with the responses being made by the other professionals as they act on the expanded object of activity. For example, a teacher may wait until a social worker has helped a family address its housing problems before making demands on the family about school attendance.

Importantly, working relationally also enhances the professional agency of practitioners, as they are not expected to tackle tasks best addressed by someone with the appropriate professional knowledge. Here are social workers in two smaller projects underway, also using DWR, over the same time period as NECF and LIW, discussing how relational work has enhanced their professional identities.

So, you always have different perspectives, and that helps you grow, I think, professionally and you have more of this community feeling, to work together towards a child's or a family's wellbeing.

It has helped me to think about what my core skills are as a social worker.

It's about sharing out of responsibilities. …you're not on your own dealing with this … Everybody has got responsibilities, everybody will go away and do their jobs and bring it back and you're not on your own. And that confidence is hugely important when you are dealing with very complex family situations that you don't feel you're isolated and on your own.

The descriptions so far sound benign and easy to achieve. But the segmentation of knowledge and values within practices can inhibit communication between different professionals. This is why discussions about motives and values, which eliminate or reduce hierarchies of knowledge and professional status, are an important part of the backstage work. A cultural‐historical approach helps with these processes as it recognizes that creative responses to problems are based in recognizing contradictions, exploring differences and expanding understandings (Engeström & Sannino, 2011).

## EMPLOYING THE CONCEPTS IN DISCOVERED PROBLEMS

The three relational concepts have now become resources for researchers who are attempting to explain forms of collaboration. Topics include relations between university research and policy‐making (Rickinson & Edwards, 2021); leadership expertise (Daniels & Edwards, 2012; Edwards & Montecinos, 2017); pedagogy in school (Hedegaard & Edwards, 2023; Nyborg et al., 2022; Shires, 2023). Here I shall discuss how they were used to explain how family workers supported vulnerable clients (Hopwood & Edwards, 2017); the design of a smartphone app to support autistic young people (Edwards & Fay, 2019) and an evaluation study (Fleer et al., 2017).

### Using the relational concepts to explain pedagogic work with clients

The examples here come from Hopwood's Sydney‐based study of work in a ‘Day and Stay’ clinic for families needing help with their children's behaviour (Hopwood & Edwards, 2017). The discovered problem was how to capture and interpret interactions between family workers and parents of very young children in order to be able to explain successful practitioner approaches. The solution was to record interactions and code the data using relational expertise, common knowledge and relational agency as ways of explaining and discussing the professional aims and actions of the practitioners.

The first extract of an interaction is from a home visit by Sophie (a nurse) to Masha (a new mother) (Hopwood & Edwards, 2017). Masha is highly anxious about her 10‐week‐old son. She is also being heavily criticized by her mother‐in‐law. Consequently, she is strongly protective of her son in ways that can inhibit his development. In the exchange that follows, Sophie and Masha are sitting together, watching the baby lying on his back on a playmat. Sophie is encouraging Masha's observations of the child.

Masha: Now he likes the playmat a lot, he gets very excited.

Sophie: What does he do to let you know he is excited?

Masha: Oh, he smiles and laughs. When I'm holding him, we just look at each other for a moment and he smiles or giggles.

Sophie: Those little stops, when you gaze together and wonder, they are like food for the brain. He's got this big, exciting world out there he's just getting to know and you're helping him connect all the stars.

Masha: Oh.

In this exchange, the object of activity is the child's behaviour. Sophie is demonstrating relational expertise by helping Masha expand her understanding of her son's actions. Together they are building common knowledge, sharing how they are interpreting and orienting towards the infant. This common knowledge then becomes a resource, which Sophie can refer to in later visits. In these subsequent visits Sophie's aim is to enable Masha to work relationally with her son to encourage his agency.

The second extract is from a visit to the centre discussed in the same article. Here too, there is dual focus on the agency of the parents and the agency of the child. Bik and her partner Lee are concerned that 9‐month‐old Rachel is refusing food that they feed her. Sarah, a family worker, has worked with Bik and Lee over Rachel's sleeping patterns and has built a sound relationship with them. The three adults are sitting together while Rachel is playing with a biscuit. Like Sophie, Sarah offers a running commentary which encourages the parents to observe the child closely, but without anxiety, focusing on what she can do.

Sarah…. She might be feeling under pressure if you're looking at her all the time…see she is exploring putting that cracker in her mouth. [Mealtime] … needs to be relaxed, no pressure, just the opportunity to explore.

Bik (to Lee): See that's what I say!

Lee: She keeps pushing it in [a large piece of cracker].

Bik: She'll work it out.

Sarah: Exactly! To work it out, she needs practice. That gag reflex is a safety mechanism.

Bik: It is probably better for her to hold when it is bigger.

Sarah: Exactly! Around nine months that pincer grip will come into place. It's all about fun time exploring food…If she has a good time she'll come back for more.

Bik: Yes, she is relaxed now (to Lee) because you are not shoving food in her mouth.

Sarah is concerned that Bik and Lee's anxieties are inhibiting the unfolding of Rachel's agency as she moves towards her second year. While addressing Rachel's sleeping patterns, Sarah had built common knowledge with Bik and Lee, recognizing their orientations towards Rachel and being explicit herself about how the parents can encourage Rachel's agency.

The relational concepts help at two levels in this analysis. At one level, we can see that Sarah is using common knowledge to work relationally alongside Bik. In brief, she is enabling the unfolding of Bik's agency as a parent. At the other level, Bik's agency relates to Sarah's concern that Rachel's agency is being restricted at mealtimes. This exchange reveals that the object of activity, now shared by Sarah and Bik, is Rachel's agentic control over feeding. These analyses suggest that there is considerable potential here for the relational concepts being used in training programmes for those working in the caring professions.

### Developing an app to support young people with autism in the workplace

Institutions such as schools and workplaces are made up of practices where the object motives embedded in the practices, such as children's academic achievement or making a profit, give shape to the practice and the motive orientations of participants. These higher order motives are translated to a micro‐level within institutional practices where they become recurrent demands: ‘this is the way we do it here’. The challenge for new entrants is to learn to recognize these demands.

Young people with an Autistic Spectrum Condition (ASC) are likely to be inflexible, very literal and to dislike ambiguity. Importantly, they may have strongly held motive orientations, noticing things other people do not notice and be oblivious to what is important for others within a practice. The discovered problem is that challenges in transitions between practices, such as entering a new workplace or moving from school to college, are amplified for such young people. Consequently, they need clear guidance about what to do when entering a new practice or when dealing with changes in a familiar practice. If one can reveal (i) their motive orientations and (ii) the demands in the practices they are entering, they can be helped to develop new motive orientations and engage with the recurrent demands in the practices.

The problem is a serious one. One UK estimate is that one in a 100 children, young people and adults have ASC (Baird et al., 2006); while a US estimate is one in 68 (Chen et al., 2015). Only 15% of adults in the UK with ASC are in full‐time employment (National Autistic Society, 2018), while the same report found that 33% of adults with ASC experience severe mental health problems due to a lack of support. The right support can make a difference. The detailed report by Wittemeyer and colleagues (Wittemeyer et al., 2011) on the outcomes for young people with ASC observed that young people felt that letting people know about their ASC diagnosis made a huge difference to how they could engage in practices.

One attempt at tackling the problem of young people with ASC in training or employment was the design of a smartphone app. Called the ‘Me at Work’ app, the intention was that it could contain the common knowledge of both the designated college tutor or employer and the young person (Edwards & Fay, 2019). The premise was that common knowledge could be made explicit and become a resource that would mediate the development of the new motive orientations demanded by a transition into a new practice. In year one, as the first step in designing the app, a small unfunded pilot study was undertaken to reveal the motive orientations of potential stakeholders. Ethical approval was gained through the approval system of the Special School that hosted the study. The following people were interviewed: three teachers in a Special School where 42% of pupils have ASC; one Further Education Tutor responsible for young people with ASC; four young people with ASC about to leave a Special School; three parents; five employers who employed people with ASC and two young people with ASC in employment undertaking data entry. The different motive orientations are shown in Table 1.

**TABLE 1:** Stakeholder motive orientations in relation to support for a young person with ASC.

Sources of ideas	The motive orientations
The educators	There is a limit on what we can do. School can help prepare the young person through developing life skills, but can not focus on specific demands in workplaces The young person and family need to have some control over the transition They should present a rounded profile showing their capabilities
The young people at school	I need to know details about the day and the workplace You need to know things about me to help me in the workplace I am good at some things
The parents	It can be helpful if parents can be part of the transition process
The employers	The young person needs to know what we expect of them in the job they will be doing We expect to see some progress over time
The young people in employment	There are things that are interesting about me outside work

The intention was that the common knowledge held in the App would be a resource for the young person as they tried to enter and contribute to the new‐to‐them practices. The App was designed so that their anxiety was reduced while they were guided to new motive orientations and competences. We know that digital technologies are liked by many young people with ASC (Chen et al., 2015; Parsons et al., 2015). The design of this App would encourage their independence because they would be in control, as it would show what they know and demystify what they do not know or understand.

**FIGURE 1:**
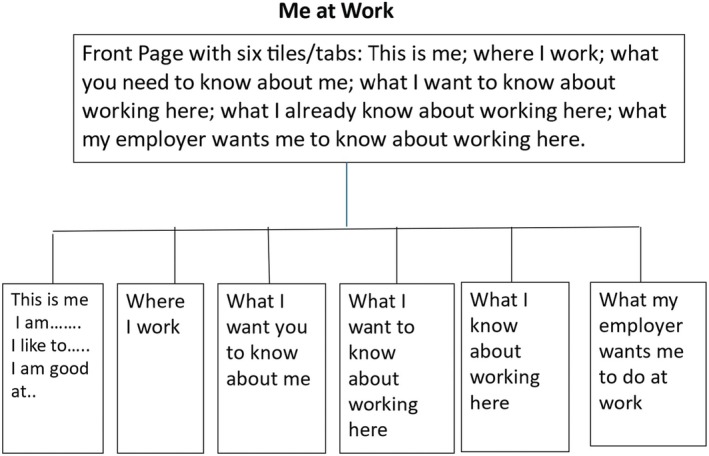
The outline of the me at work app.

The plan for the App is shown in Figure 1. The young person, perhaps with a parent's help, would complete the first, third, fourth and fifth tiles. The employer would complete and update the second and sixth tile via an email to the young person and parent. The design would be supported by sets of suggestions for completing each tile.

The App was to have been piloted in year two and rolled out in year three. Unfortunately, the company that was to make the App was reorganized and no longer willing to undertake pro bono work. Hence, the resource remains at the design stage, ready to be developed by those who can access the funding unavailable to an Emerita. The design has been discussed with specialist colleagues and in a seminar with practising Educational Psychologists, who recognized the potential in the design principles for supporting the transitions of students with different needs in school, further education and higher education.

### Creating an evaluation resource: The relational agency framework

Fleer and colleagues at Monash University were asked to evaluate an initiative aiming at developing strong interprofessional working among early years practitioners from education, health and welfare across the State of Victoria. However, the programme, which involved 132 workers meeting in local networks, was already underway when the team was commissioned. Consequently, the researchers required a way of categorizing a large amount of existing paper‐based evaluation comments, reflective essays and questionnaires from each network, none of which they had designed.

For the purposes of the evaluation, the categorization needed to capture a network's progress towards interprofessional collaboration. The team's solution to this discovered problem was to draw on the three relational concepts to create the Relational Agency Framework (RAF) (Fleer et al., 2017).

**FIGURE 2:**
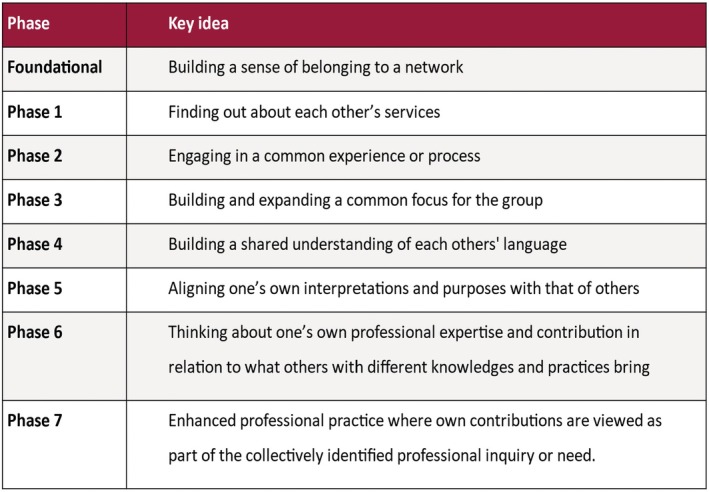
The relational agency framework.

The RAF (Figure 2) was designed as a tool to assess the maturity of a network in terms of shifts towards collaboration across practice boundaries. For example, at Phase 5, participants discussed the outcomes of their work as specialist practitioners and supported each other's analyses and reflections with some understanding of the other practice. While in mature networks at Phase 7, a shared understanding was in place and was evident in the multidisciplinary narratives that were offered (Duhn et al., 2016).

The research team described the RAF as resource ‘for supporting the establishment, maintenance and development of a network for multidisciplinary groupings of practitioners.’ (Fleer et al., 2017, p. 223). The framework was subsequently adopted by the Victoria Department of Education to support the development of multi‐professional collaborations in other school sectors.

## DISCUSSION AND IMPLICATIONS

The intention is that the analyses presented here speak to practicing psychologists and those in related professions, and to researchers, and indeed to those who combine both practitioner and researcher roles. The three relational concepts, relational expertise, common knowledge and relational agency, label the processes that were identified while observing collaborations. These collaborations drew on the strengths of all participants, including children and families, and led to their agentic actions in and on the practices they inhabited. The collaborations were across practice boundaries and were not based in studies of established teams, where people were familiar with the professional motives and strengths of colleagues. Nonetheless, they are a useful resource for examining how teams are working, whether one profession dominates or another is silenced.

The labels were intended to guide training programmes that tackled interprofessional working and mark a move from those courses which saw interprofessional work as a matter of producing the generalist practitioner. Instead, the three relational concepts require participants to identify their own expertise and to elicit and respect the expertise of others. This emphasis on specific expertise also argues against any notion of good relational expertise as a substitute for the special insights, based on training and/or experience, that participants bring to collaborations.

As the discussions of the discovered problems have shown, the three relational concepts have also provided researchers with analytic resources when trying to capture how practitioners build the agency of clients, design resources to support vulnerable clients or evaluate progress towards strong collaborations among people who work together fleetingly on a variety of different problems. These analyses also reveal how a cultural‐historical focus on actions in practices allows, as the daycare centre study showed, researchers to track changes over time.

Above all, the three relational concepts offer a shared way of understanding those collaborative processes that lead to an expansion of a problem, so that more of its complexity is revealed and the widest array of possible responses are brought into play. Ultimately, the concepts have the potential to guide the strengthening of children and their families. Labelling and explaining the concepts and their intentions, to inform collaborations in this way, reflects Wardekker's view that: ‘Knowledge is a mediational means for focusing our attention on specific aspects of practice.’ (Wardekker, 2000, p. 269) together with Engestrom's observation that: ‘My key message is that forming theoretical concepts is more important than ever in our age of chaotic complexity and crises’ (Engeström, 2020, p. 32). My intention has been to explain and illustrate the relational concepts in different practices, in the hope that might inform the work of psychologists and others in the broad fields of education and welfare.

## AUTHOR CONTRIBUTIONS

**Anne Edwards:** Conceptualization; investigation; funding acquisition; methodology; validation; writing – review and editing; formal analysis; project administration; data curation; supervision; resources; writing – original draft.

## FUNDING INFORMATION

Learning in and for Interagency Working: ESRC RES‐139‐25‐01; National Evaluation of the Children's Fund: DFES, RR 734.

## CONFLICT OF INTEREST STATEMENT

There are no conflicts of interest.

## Data Availability

Data sharing is not applicable to this article as no new data were created or analyzed in this study.
